# The higher levels of self-reported satisfaction, the lower risk of depressive symptoms: Evidence from a nationwide cross-sectional study in China

**DOI:** 10.3389/fmed.2022.844964

**Published:** 2022-09-20

**Authors:** Zhiping Niu, Feng Zhao, Weihong Wen, Donghui Han, Keying Zhang, Xiaolong Zhao, Shichao Han, Fa Yang, Zhizhou Duan, Weijun Qin

**Affiliations:** ^1^Department of Urology, Xijing Hospital, The Fourth Military Medical University, Xi'an, China; ^2^Department of Anesthesiology, Heze Municipal Hospital, Heze, China; ^3^Institute of Medical Research, Northwestern Polytechnical University, Xi'an, China; ^4^Preventive Health Service, Jiangxi Provincial People's Hospital, The First Affiliated Hospital of Nanchang Medical College, Nanchang, China

**Keywords:** depressive disorder, satisfaction, middle-aged and older adults, mental health, China

## Abstract

**Objectives:**

This study aimed to investigate the associations between several dimensions of self-reported satisfaction and the risk of depressive symptoms among Chinese middle-aged and older adults.

**Methods:**

The China Health and Retirement Longitudinal Study (CHARLS) conducted a nationwide cross-sectional study of middle-aged and older adults. Depressive status was evaluated using the 10-item center for epidemiological studies depression scale (CESD-10), and self-reported life, health, marital status, parent-child relationship, and air quality satisfaction were adopted using Likert 5-point evaluation methods. A generalized linear model (GLM) was applied to explore the association between satisfaction and depression risk.

**Results:**

A total of 13,978 Chinese people aged over 45 years old were included in this study, and 35.7% of the participants had depressive symptoms. The GLM analysis indicated that all dimensions of satisfaction were negatively associated with the risk of depressive symptoms. For each 1-point increase in life, health, marital status, parent-child relationship, and air quality satisfaction, the incidence of depressive symptoms decreased by 60.8% (odds ratio (OR) = 0.392; 95% confidence interval (CI): 0.370, 0.414), 56.3% (OR = 0.437; 95% CI: 0.418, 0.458), 41.8% (OR = 0.582; 95% CI: 0.555, 0.610), 37.2% (OR = 0.628; 95% CI: 0.596, 0.662), and 25.6% (OR = 0.744; 95% CI: 0.711, 0.778), respectively.

**Conclusion:**

Higher satisfaction levels with life, health, marital status, parent-child relationship, and air quality are associated with a lower risk of depressive symptoms among middle-aged and older adults. Given the aging society and the increasing mental health problems of middle-aged and older adults in China, our study provides a comprehensive perspective for depression prevention and mental health improvement.

## Introduction

Depression, one of the most common mental disorders, affects more than 264 million people worldwide (1, 2) and leads to more than 50 million Years Lived with Disability from 1990 to 2017(3). Depressive disorder often results in cognitive impairment, sleep disorders, dementia, and even suicide, causing a serious burden to the individual, family, and society (4–6). Several epidemiological studies demonstrated that people who experience depressive symptoms might have an increased risk of a variety of illnesses, disabilities, and death, even without a formal diagnosis (1, 7). Moreover, without efficient intervention for existing depressive symptoms, undiagnosed depression symptoms might lead to progression and a more serious disease burden (8).

China has experienced an aging society since the end of the 20th century, with the aging trend taking a toll on public healthcare systems. According to the seventh National Census of China, people aged 65 years or above accounted for 13.50% of China's population in 2020. The proportion of the elderly population has increased by 4.63% in the last decade (9). A meta-analysis published in 2018 suggested that about 2.7% of elderly adults have suffered from a major depressive disorder (10). Moreover, middle-aged adults are rapidly entering the ranks of the elderly. The middle-aged population is also suffering from decreased mental health (11). For example, Choi et al. conducted a longitudinal study in Korea and found that 35.1% of middle-aged adults (aged from 45 to 54 years old) had depressive symptoms (12). Given the aging society and increasing mental health problems of middle-aged and older adults, identifying risk factors for depression is of great significance for mental health promotion in the middle-aged and elderly populations in China.

Mental health might be affected by a combination of individual, family, and environmental factors, and it is not easy to find a comprehensive indicator covering all aspects of those factors (13–15). Satisfaction is a comprehensive psychological index to evaluate the difference between expectation and perception. Several studies reported that many satisfaction aspects are associated with mental health. For example, a Canadian national survey suggested that life satisfaction was strongly associated with self-reported mental health (16). Marital relationships could also affect people's mental health. A study involving 728 women suggested that greater marital satisfaction was associated with fewer depression symptoms (17). The relationship between parents and their children may also affect the parents' mental health. For example, Chen et al. showed that parent-child relationship satisfaction was negatively associated with depressive symptoms and diagnosed depression (18). Finally, air quality and health conditions might be important factors for mental health. Although the associations between air quality satisfaction, health satisfaction, and depression may not have been examined, numerous studies suggested that people experiencing higher levels of air pollution (16–18) and worse health conditions (19, 20) are more likely to suffer from depression and other mental health problems. Based on the abovementioned studies, we proposed a hypothesis that satisfaction with life, health, marital status, parent-child relationship, and air quality might be associated with depressive status.

**Figure 1:**
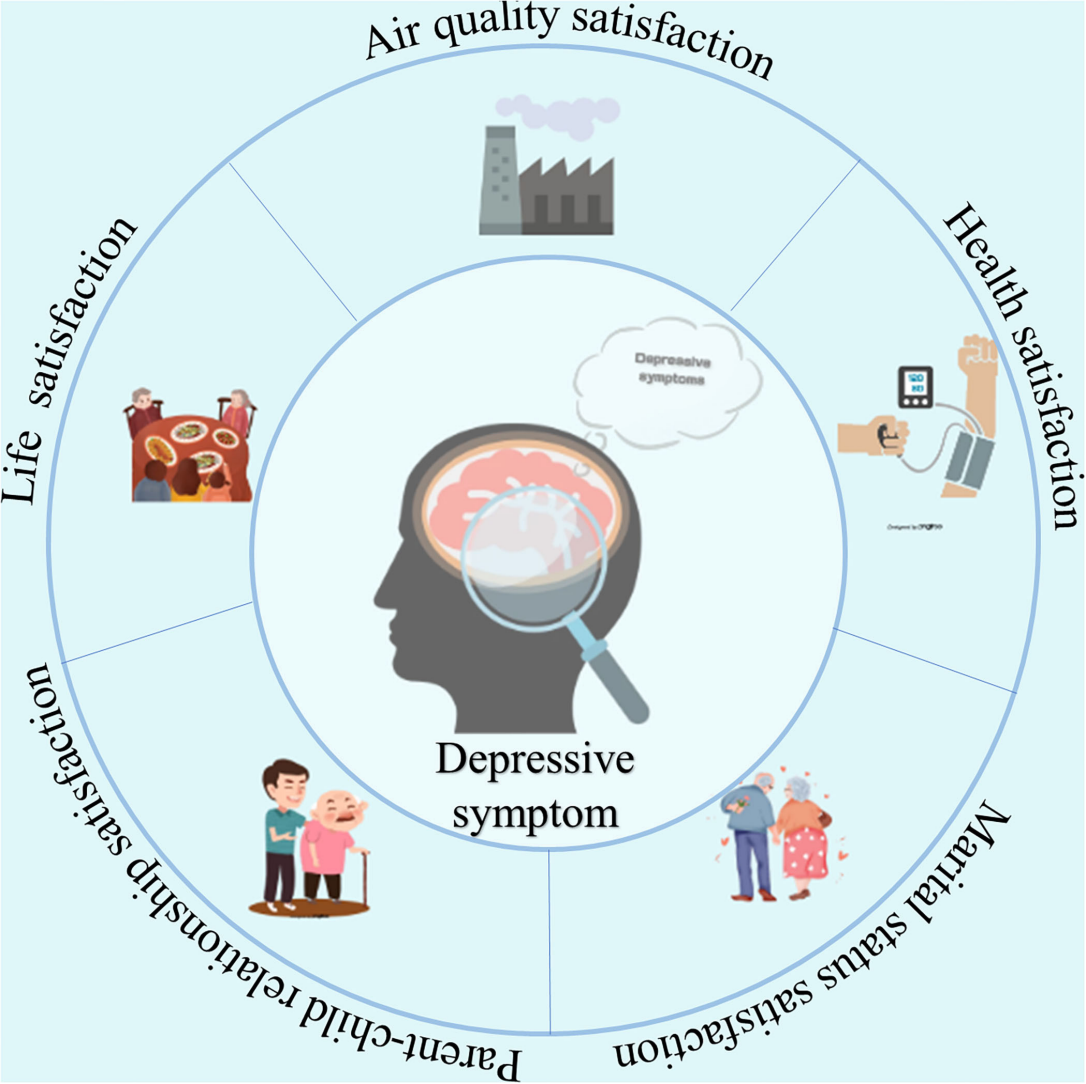
The research hypothesis of the present study.

To the best of our knowledge, no study has comprehensively explored the effects of satisfaction with life, health, marital status, parent-child relationship, and air quality on depressive symptoms. To fill this gap, the present study aimed to explore the relationship between five dimensions of self-reported satisfaction and depressive symptoms. The research hypothesis of our study is shown in Figure 1. We focused on (1) the prevalence of depressive symptoms among the Chinese middle-aged and elderly populations; (2) the associations between each aspect of satisfaction and risk of depressive symptoms; and (3) whether these relationships differ among populations with different characteristics.

## Methods

### Study population

The study population was based on the China Health and Retirement Longitudinal Study (CHARLS) wave 4 in 2018. CHARLS is a national cohort study focused on Chinese residents aged 45 and older in China, which covers 150 county-level cities in 28 provinces of China and has collected a set of high-quality microdata of middle-aged and elderly adults. A multi-stage stratified probability proportionate to size sampling method was employed to recruit participants. A structured questionnaire was used to collect their health status and other relevant information *via* face-to-face interviews (21). Previous studies described detailed information (5, 22, 23) and are available at the CHARLS website (http://charls.pku.edu.cn/). A total of 19,816 participants from 450 communities in 28 provinces of China were investigated in wave 4, with an 83.84% response rate.

The Institutional Review Board approved the study protocol of CHARLS at Peking University (Code: IRB00001052-11015), and informed consent was obtained from all participants.

### Assessment of depressive symptoms

The 10-item center for epidemiological studies depression scale (CESD-10) was applied to assess the depression status of middle-aged and elderly adults, which was developed from the 20-item CESD version and has been adopted widely in population-based depression status studies (24, 25). Briefly, participants were asked about their feelings and behaviors during the last week. Each negative item (items 1–4, 6,7,9, and 10) was scored as 3 (most or all of the time (5–7 days)), 2 (occasionally or a moderate amount of the time (3–4 days)), 1 (some or a little of the time (1–2 days)), and 0 (rarely or none of the time (<1 day)), while two positive items (items 5 and 8) were scored as 0 (most or all of the time (5–7 days)), 1 (occasionally or a moderate amount of the time (3–4 days)), 2 (some or a little of the time (1–2 days)), and 3 (rarely or none of the time (<1 day)) (26). To make the questionnaire readable, all items of CESD-10 were provided in the English-Chinese edition (Supplementary Table S1). The 10-item CESD total score ranges from 0 to 30, and a higher CESD total score indicates a more severe depression status. To evaluate the effects of self-reported satisfaction on depression in Chinese middle-aged and elderly adults, we recognized respondents as having depressive symptoms group if their CESD score was ≥ 10, while participants were categorized into the non-depressive symptoms group when their CESD score was <10 (5, 25). The Cronbach's alpha coefficient was 0.803.

### Satisfaction assessment

There are many dimensions to people's satisfaction that might affect their health. In the present study, we used five dimensions of satisfaction: life satisfaction, health satisfaction, marital status satisfaction, parent-child relationship satisfaction, and air quality satisfaction. Each dimension of satisfaction was assessed independently using face-to-face interviews using a Likert five-point evaluation method (Supplementary Table S2). Briefly, the participants were asked to rate “How satisfied are you with your life/health/ marriage/relationship with children/air quality?.” Each item was assessed using a five-point scale: 5 (completely satisfied), 4 (very satisfied), 3 (somewhat satisfied), 2 (not very satisfied), and 1 (not at all satisfied). A higher reported score represented better satisfaction. The Cronbach's alpha coefficient was 0.700. The Kaiser–Meyer–Olkin (KMO) value was 0.759, and the difference of Bartlett's Sphericity test was significant (χ^2^ value = 11621.023, *P* <0.001). The detailed questionnaire in the English-Chinese edition is shown in Supplementary Table S2.

### Covariates

Numerous potential modifications were considered in the analysis. The sociodemographic variables included age, sex (male *vs*. female), residence (urban *vs*. rural), education level, and marital status. Educational level was categorized into low (elementary school or below), moderate (middle, high, vocational school, and associate degree), and high (college and above). Marital status was classified into three groups (married and living with spouse *vs*. married but living without spouse *vs*. single, divorced, or widowed) (26). Health behavior variables included smoking status (non-smoking *vs*. smoking) and alcohol drinking status (non-drinking *vs*. drinking). In addition, we added regional variables (East vs. Middle vs. West) to the analysis to consider the impact of regional and economic development levels.

### Statistical analysis

Pearson's correlation test was used to examine correlations among different dimensions of satisfaction. A generalized linear model (GLM) was employed to examine the associations between self-reported satisfaction and depressive symptoms. The effect estimates for all five dimensions of satisfaction were reported as odds ratios (OR) and their 95% confidence intervals (95% CIs) of depressive symptoms per 1-point increase in satisfaction score. Three models were used. First, the crude model without any potential confounders was developed (Model 1). Then, sociodemographic variables (age, sex, educational level, and marital status) were included in the model as covariates (Model 2). Finally, Model 3 (fully adjusted model) was additionally adjusted for health behavior (smoking and drinking status) and regional differences.

We also examined whether the associations between self-reported satisfaction and depressive symptoms were potentially modified by age, sex, residence, education level, marital status, smoking status, drinking status, and regional categories. Subgroup analyses of each potential modifier were performed by adding a cross-product term into the fully adjusted model (Model 3) (26).

All analyses were conducted using R software version 4.0.3, and a *p-*value <0.05 was regarded as statistical significance.

## Results

**Figure 2:**
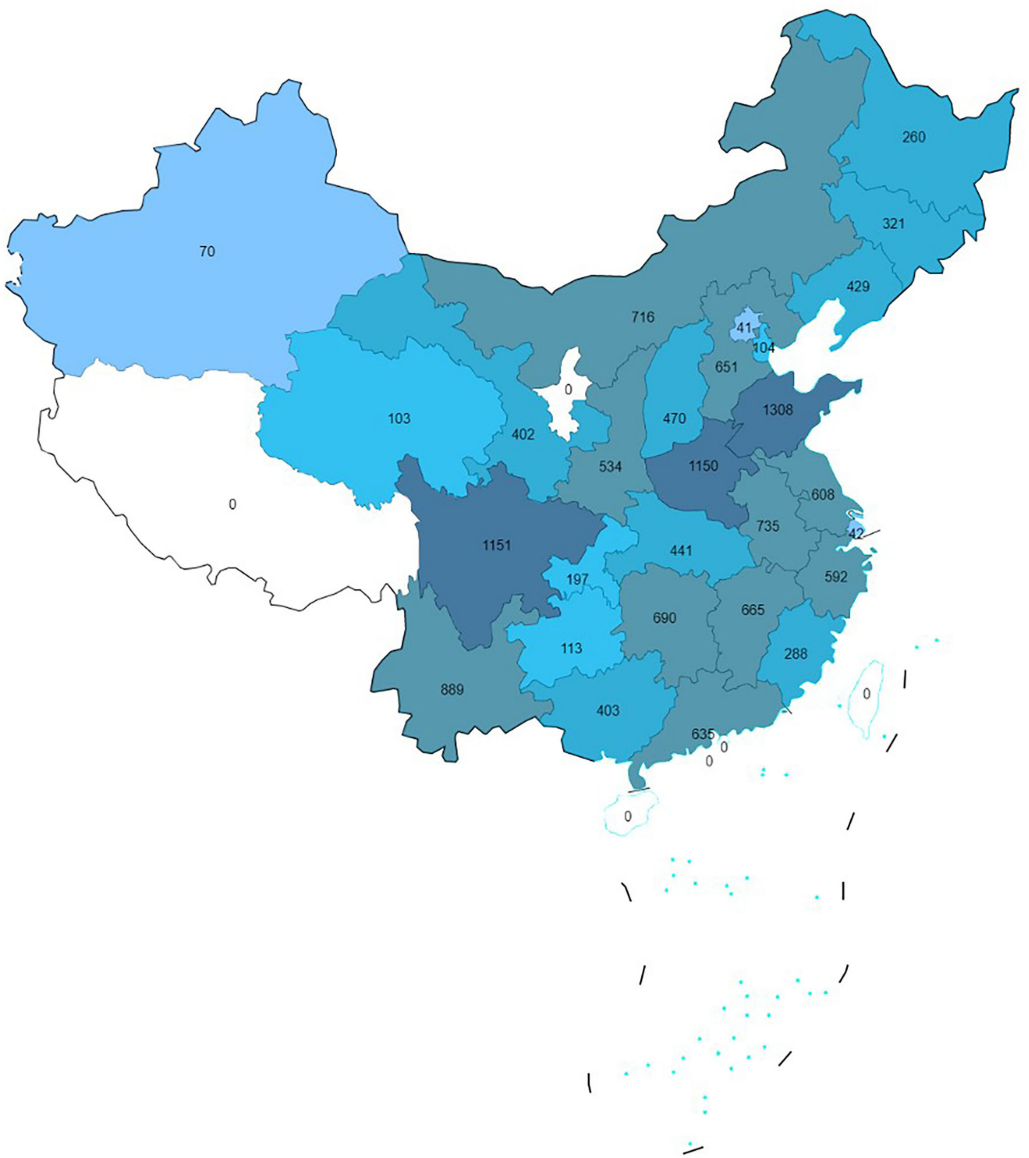
Geographical distribution of the participants.

After excluding 5,791 participants with a missing depressive score or satisfaction assessment data, and 47 participants without vital demographic characteristics, a total of 13,978 middle-aged and elderly adults were included in our study. The sample range covered 126 county-level cities in 28 provinces of China, and the distribution of participants is presented in Figure 2. The mean age of participants was 60.48 ± 18.82 years. Among them, 7,012 (50.2%) participants were men. Most of the participants lived in rural areas (59.6%), reported low (elementary school or below) education levels (61.2%), were married and living with a spouse (89.0%), and were categorized as non-smokers (93.5%) and non-drinkers (63.3%). Moreover, 36.2% of the participants were recruited from East China, 38.8% were from Middle China, and 25.0% were from West China. A total of 4,986 participants scored 10 points and above, giving a prevalence of depressive symptoms of 35.7%. More detailed information regarding the participants is shown in Table 1.

**Table 1:** Basic characteristics of participants in the China Health and Retirement Longitudinal Study (CHARLS) wave 4.

**Characteristics^a^**	**Total (*n* = 13,978)**	**Non-depression symptoms (*n* = 8,992)**	**Depression symptoms (4,986)**	***P*-value**
Age, years	60.48 ± 18.82	60.15 ± 9.01	61.08 ± 29.09	0.427
Satisfaction				
Life satisfaction	3.28 ± 0.77	3.45 ± 0.67	2.97 ± 0.84	<0.001
Health satisfaction	2.97 ± 0.90	3.19 ± 0.79	2.58 ± 0.95	<0.001
Marital satisfaction	3.43 ± 0.81	3.56 ± 0.73	3.19 ± 0.90	<0.001
Children's relationship satisfaction	3.64 ± 0.71	3.72 ± 0.67	3.50 ± 0.76	<0.001
Air quality satisfaction	3.16 ± 0.82	3.21 ± 0.80	3.07 ± 0.85	0.014
Gender				<0.001
Male	7012 (50.2)	4989(55.48)	2023(40.57)	
Female	6966 (49.8)	4003(44.52)	2963(59.43)	
Residence				<0.001
Rural	8328(59.6)	4965(55.22)	3363(67.45)	
Urban	5650(40.4)	4027(44.78)	1623(32.55)	
Educational level				<0.001
Elementary school or below	8554(61.2)	4994(55.54)	3560(71.40)	
Middle, High, Vocational school and Associate degree	5312(38.0)	3901(43.38)	1411(28.30)	
College and Above	112(0.8)	97(1.08)	15(0.30)	
Marital status				<0.001
Married and living with spouse	12442(89.0)	8118(90.28)	4324(86.72)	
Married but living without spouse	996(7.1)	582(6.47)	414(8.30)	
Single, divorced, and windowed	540(3.9)	292(3.25)	248(4.97)	
Smoking Status^b^				<0.001
Non-smoker	7833(93.5)	4783(92.93)	3050(94.43)	
Smoker	544(6.5)	364(7.07)	180(5.57)	
Drinking status^b^				<0.001
Non-drinker	8849(63.3)	5358(59.64)	3491(70.13)	
Drinker	5113(36.6)	3626(40.36)	1487(29.87)	
Regional categories				<0.001
East	5059(36.2)	3635(40.42)	1424(28.56)	
Midland	5418(38.8)	3410(37.92)	2008(40.27)	
West	3501(25.0)	1947(21.65)	1554(31.17)	

^a^ For continuous variables, numbers represent the mean ± standard deviation and for categorical variables, numbers represent count (percentage). ^b^ For variables, missing values existed. P-values represented the difference test between non-depression symptoms group and depression symptoms group.

Table 2 presents the average score of all five dimensions of self-reported satisfaction and depressive status. The mean levels of life, health, marital status, parent-child relationship, and air quality satisfaction were 3.28 ± 0.77, 2.97 ± 0.90, 3.43 ± 0.81, 3.64 ± 0.71, and 3.16 ± 0.82, respectively. In terms of depressive status, the mean CESD-10 score was 8.24 ± 6.39. Pearson's correlation test suggested that a high correlation existed among different satisfaction dimensions (Supplementary Table S3).

**Table 2:** Self-reported satisfaction and depression score of participants.

**Variable**	**Mean**	**SD**	**P25**	**P50**	**P75**	**IQR**
Satisfaction						
Life satisfaction	3.28	0.77	3.00	3.00	4.00	1.00
Health satisfaction	2.97	0.90	2.00	3.00	4.00	2.00
Marital satisfaction	3.43	0.81	3.00	3.00	4.00	1.00
Children's relationship satisfaction	3.64	0.71	3.00	4.00	4.00	1.00
Air quality satisfaction	3.16	0.82	3.00	3.00	4.00	1.00
Depressive status						
CESD-10 score	8.24	6.39	3.00	7.00	12.00	9.00

SD, standard deviation; IQR, inter-quartile range; CESD-10, the 10-item center for epidemiologic studies depression scale.

**Figure 3:**
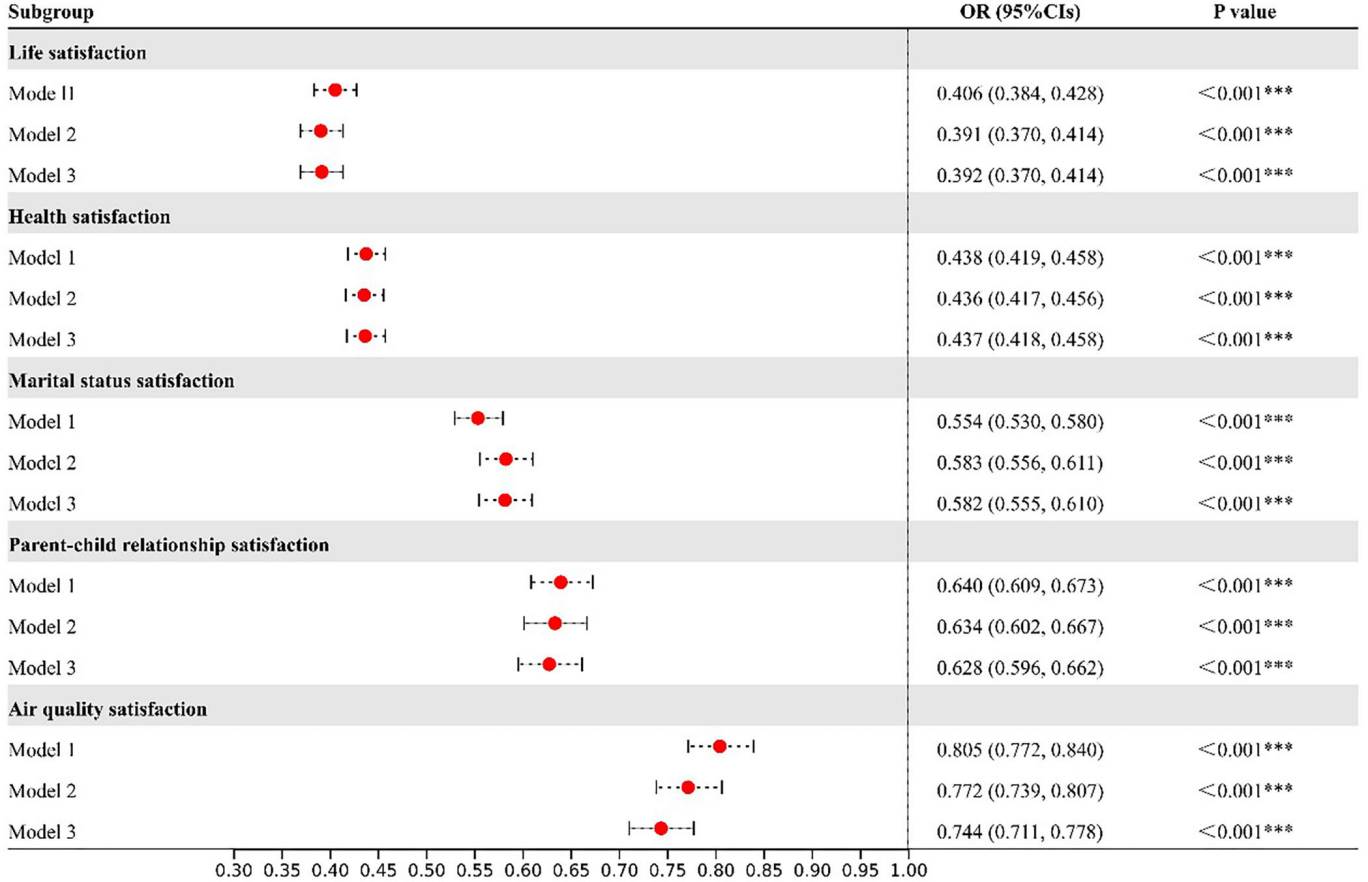
Associations between each 1-point increase in satisfaction and the odds ratio (with 95% confidence intervals (CIs)) of depressive symptoms. Model 1: Crude model. Model 2: Adjusted for age, gender, educational level, and marital status. Model 3: Adjusted for age, gender, educational level, marital status, smoking status, drinking status, residence, and regional categories.

Figure 3 presents the associations between all five dimensions of satisfaction and the risk of depressive symptoms. Higher levels of life, health, marital status, parent-child relationship, and air quality satisfaction were all strongly related to a lower risk of depressive symptoms in model 1. When considering the potential confounders in the models, the results were still significant for these five dimensions of satisfaction (Model 2 and Model 3). After adjusting for sociodemographic factors, health behavior factors, and regional differences, we found that each 1-point increase in life, health, marital status, parent-child relationship, and air quality satisfaction was associated with 60.8% (OR = 0.392; 95% CI: 0.370, 0.414), 56.3% (OR = 0.437; 95% CI: 0.418, 0.458), 41.8% (OR = 0.582; 95% CI: 0.555, 0.610), 37.2% (OR = 0.628; 95% CI: 0.596, 0.662), and 25.6% (OR = 0.744; 95% CI: 0.711, 0.778) decrease in the risk of depressive symptoms, respectively (Figure 3).

**Figure 4:**
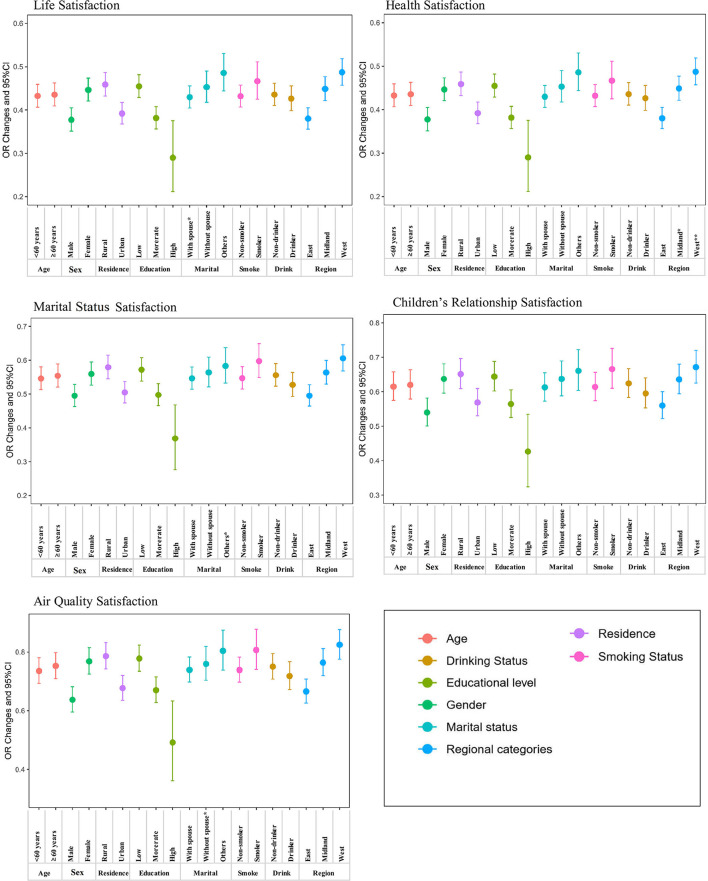
Stratified analysis of the association of depressive symptoms with every 1-point increase in satisfaction.

Figure 4 and Supplementary Table S4 show the associations between self-reported satisfaction and depressive symptoms, stratified by age, sex, residence, education level, marital status, smoking status, drinking status, and regional categories. Participants younger than 60 might be more susceptible to depressive symptoms than the others, although there was mostly no statistical significance. For example, each 1-point increase in life satisfaction was associated with a 61.6% (OR = 0.384, 95% CI: 0.357, 0.413) decrease in depressive symptoms among people aged <60 years old, while this effect estimate was 59.7% (OR = 0.403, 95% CI: 0.375, 0.423) in people aged 60 years old or above. Moreover, our results suggested that participants with different residence, educational levels, marital status, and regional categories might have different effect estimates in some dimensions of satisfaction. For example, life satisfaction had a stronger effect on depressive symptoms among urban residents (OR = 0.356, 95% CI: 0.331, 0.384) than that of rural residents (OR = 0.414, 95% CI: 0.385, 0.445). Compared with participants with an elementary school or below educational level (OR = 0.408, 95% CI: 0.380, 0.438), people with a college degree and above were more sensitive to life satisfaction (OR = 0.253, 95% CI: 0.186, 0.326). Marital status might also modify the relationship between marital satisfaction and depressive symptom risk. Compared with participants who were married and living with a spouse (OR = 0.547, 95% CI: 0.514, 0.580), single people, divorced, or widowed were less susceptible to depression symptoms (OR = 0.583, 95%CI:0.533, 0.637). In addition, we found that the regional category might interact with life satisfaction and health satisfaction. For example, compared with people lived in East China (OR = 0.380, 95% CI: 0.356, 0.405), people living in Middle China (OR = 0.449, 95% CI: 0.422, 0.477) or West China (OR = 0.487, 95% CI: 0.457, 0.519) had a greater effect on people's health satisfaction.

## Discussion

This large-scale nationwide study suggested that the prevalence of depressive symptoms was 35.7% among Chinese middle-aged and elderly populations. Higher levels of life, health, marital status, parent-child relationship, and air quality satisfaction were related to a lower risk of depressive symptoms. For each 1-point increase in life, health, marital status, parent-child relationship, and air quality satisfaction, the risk of depressive symptoms would decrease by about 60.8, 56.3, 41.8, 37.2, and 25.6%, respectively. Additionally, we observed that participants who were aged <60 years old, were men, lived in an urban area, had higher-level education, were married and living with a spouse, did not smoke, drank alcohol, and were from East China might be more vulnerable to the risk of depressive symptoms than the others for several satisfaction dimensions.

Several epidemiological studies also examined the association between depressive status and individual satisfaction dimensions, mainly aimed at life satisfaction and marital satisfaction. As a composite measure of satisfaction, life satisfaction has been proven to have good psychometric properties and is associated with depressive status. For example, a cross-sectional study conducted in Poland showed that higher levels of life satisfaction were related to lower levels of depression in older adults (27). Similar results were observed among elderly Chinese. A cross-sectional study of 590 empty-nest and non-empty-nest Chinese elderly adults showed that depression was the strongest predictor of life satisfaction, even compared with physical health (28). Similar associations were reported in marital satisfaction research (29–31). A cross-sectional study investigating 141 infertile couples in Iran indicated that marital satisfaction was related to depression in both male and female participants (29). In terms of parent-child relationships, a cross-sectional study of middle-aged children and their fathers in the US, which used the data from the Family Exchanges Study (FES), found that lower satisfaction regarding their relationships with their children was associated with a higher risk of depressive symptoms (32). These associations between life, marital status, and parent-child relationship satisfaction might be explained by their associations with emotional support, family function, social capital, and primary care support (33–38).

Although we did not find any direct evidence for air quality and health satisfaction, numerous epidemiological studies suggested that people experiencing a higher level of air pollution exposure and worse health conditions were more likely to be depressed. For example, a meta-analysis conducted using five studies found that each 10-μg/m^3^ increase of PM_2.5_ exposure (particulate matter with a diameter <2.5μm) was associated with a 10.2% increased risk of depression (OR = 1.102, 95% CI: 1.023, 1.189) (39). Similar results were observed for depressive symptoms. A cross-sectional study in middle-aged and elderly adults found that the incidence of depressive symptoms increased by 9% (OR = 1.09, 95% CI: 1.05, 1.14) for every 10-μg/m^3^ increase in PM_2.5_ exposure (26). The mechanisms by which air quality affects depressive status might be that air pollutants would lead to oxidative stress, immune changes, dopamine depletion, and autonomic nervous disorders, resulting in depression (40, 41). In terms of health conditions, some studies showed that people with a disease suffer a higher risk of developing a depressive disorder. For example, a British study reported a positive association between depression and ischemic heart disease risk among male adults (42). On the one hand, a depressive disorder could lead to inflammation, changes to platelet pathways, and autonomic nervous dysfunction, causing an increased risk of other diseases (34, 43, 44). On the other hand, people with other diseases are also likely to smoke and drink alcohol and often do not cooperate with medical treatments, leading to a depressive disorder (43, 45).

In the stratified analysis, it is worth noting that all five dimensions of satisfaction had a consistent trend in depressive symptoms among people with different characteristics, even if there was no statistical significance. For example, we noticed that people younger than 60 years old were more sensitive to depressive symptoms than people aged 60 years old or above. We hypothesized that this might be because people aged <60 years old would take on more family responsibilities and have higher working pressure before retirement. Consistent with the results of our study, a meta-analysis that pooled 18 studies showed that the prevalence of depression was significantly higher among older urban adults than older rural adults (46). However, considerable heterogeneities existed among the included studies, and several suggested that people in rural areas suffer a higher risk of depression (47, 48). Further studies should be conducted to explore China's urban-rural differences in depressive status. In terms of the effect value, we noticed that participants with a higher education level have significantly higher effects in the associations between all five aspects of satisfaction and the risk of depressive symptoms. A potential explanation is that more educated people might have better medical care and financial support, which could attenuate the effects of satisfaction on the risk of depressive symptoms (49, 50). Moreover, we observed that the effects of participants from East, Middle, and West China decreased in sequence, which might be explained by the fact that people in more developed areas may suffer higher life and work pressures.

Living in a society, people's mental health might be affected by a combination of individual, family, and environmental factors. Our study explored the effects of five dimensions of satisfaction on depressive symptoms, which could be classified as one comprehensive dimension (life satisfaction), one individual dimension (health satisfaction), two household dimensions (marital status and parent-child relationship satisfaction), and one environmental dimension (air quality satisfaction). Our study might provide a more comprehensive understanding of depressive conditions and their risk factors. The results for participants enrolled from 126 county-level cities in 28 provinces of China could better reflect the real depressive condition of Chinese middle-aged and elderly adults. However, several limitations of this study should be mentioned. First, depressive status and satisfaction were assessed using self-reported data. Thus, the results only reflect mental health to some extent and are not equivalent to clinically diagnosed measures. However, the CESD-10 has been adopted widely in population-based depression status investigations, and the self-reported data allow the participants to answer survey questions according to their most contributed experiences to depressive status (49). Second, we examined the associations between five dimensions of satisfaction and depressive symptoms; however, other dimensions of satisfaction, such as security and job satisfaction, need further discussion. Third, demographic characteristics, smoking, and drinking behaviors of the participants were collected using a self-reported questionnaire; therefore, reporting bias, recall bias, and misclassification might exist in our study. Fourth, the cross-sectional design is inadequate to determine causal relationships between different dimensions of satisfaction and the risk of depressive symptoms. Longitudinal studies, such as cohort studies, would be expected to confirm our results. Finally, the findings of the stratified analysis need to be taken with caution because there was mostly no statistical significance. Further studies are needed to verify our findings.

## Conclusion

This study demonstrated that more than a third of Chinese middle-aged and older adults had developed depressive symptoms. High levels of satisfaction with life, health, marital status, parent-child relationship, and air quality were associated with decreased risk of depressive symptoms in Chinese middle-aged and older adults. Given the aging society and increasing mental health problems of middle-aged and older adults, our findings provide a comprehensive basis for depression prevention and mental health improvement.

## Data availability statement

Publicly available datasets were analyzed in this study. This data can be found here: http://charls.pku.edu.cn.

## Ethics statement

The studies involving human participants were reviewed and approved by the Institutional Review Board at Peking University (Code: IRB00001052-11015). The patients/participants provided their written informed consent to participate in this study.

## Author contributions

ZN, FZ, and ZD contributed to the conception and design of the study. ZN, FZ, SH, and FY conducted the experiments and organized the database. ZN, ZD, and XZ performed the statistical analysis. ZN and FZ wrote the first draft of the manuscript. WW, DH, KZ, ZD, and WQ revised the manuscript. All authors have approved the final version. All authors contributed to manuscript revision, read, and approved the submitted version.

## Funding

This work was supported by grants from the National Natural Science Foundation of China (Nos. 81772734; 82102322) and the Innovation Capability Support Program of Shaanxi (No. 2020PT-021).

## Conflict of interest

The authors declare that the research was conducted in the absence of any commercial or financial relationships that could be construed as a potential conflict of interest.

## Publisher's note

All claims expressed in this article are solely those of the authors and do not necessarily represent those of their affiliated organizations, or those of the publisher, the editors and the reviewers. Any product that may be evaluated in this article, or claim that may be made by its manufacturer, is not guaranteed or endorsed by the publisher.
