# Supplementary material for: The higher levels of self-reported satisfaction, the lower risk of depressive symptoms: Evidence from a nationwide cross-sectional study in China
Source: Front Med (Lausanne). 2022 Sep 20;9:844964. doi: 10.3389/fmed.2022.844964 (PMC9530607; doi:10.3389/fmed.2022.844964)
Supplement: Supplementary file 1 [file Data_Sheet_1.docx]

**Supplementary File**

**Table S1. The 10-item center for epidemiologic studies depression scale (CESD-10) for assessments of depression status**

INTRO: The 10 items below refer to how you have felt and behaved during the last week. Every item has the same selective answers including rarely or none of the time, some, occasionally, and most or all of the time. Choose the appropriate response.访员指导语：下面 10 道问题是有关您上周的感觉及行为，每道题目的答案都是一样的，包括很少或者根本没有，不太多，有时或者说有一半的时间还是大多数的时间，请您选择合适的答案。

| **Item 1. I was bothered by things that don’t usually bother me 我因一些小事而烦恼。** |
| --- |
| 1. Rarely or none of the time 很少或者根本没有(<1 day 天) |
| 2. Some or a little of the time 不太多(1-2 days 天) |
| 3. Occasionally or a moderate amount of the time 有时或者说有一半的时间(3-4 days天) |
| 4. Most or all of the time 大多数的时间(5-7 days 天) |
| 8. DK 不知道 |
| 9. RF 拒绝回答 |
| **Item 2. I had trouble keeping my mind on what I was doing 我在做事时很难集中精力。** |
| 1. Rarely or none of the time 很少或者根本没有(<1 day 天) |
| 2. Some or a little of the time 不太多(1-2 days 天) |
| 3. Occasionally or a moderate amount of the time 有时或者说有一半的时间(3-4 days天) |
| 4. Most or all of the time 大多数的时间(5-7 days 天) |
| 8. DK 不知道 |
| 9. RF 拒绝回答 |
| **Item 3. I felt depressed 我感到情绪低落。** |
| 1. Rarely or none of the time 很少或者根本没有(<1 day 天) |
| 2. Some or a little of the time 不太多(1-2 days 天) |
| 3. Occasionally or a moderate amount of the time 有时或者说有一半的时间(3-4 days天) |
| 4. Most or all of the time 大多数的时间(5-7 days 天) |
| 8. DK 不知道 |
| 9. RF 拒绝回答 |
| **Item 4. I felt everything I did was an effort 我觉得做任何事都很费劲。** |
| 1. Rarely or none of the time 很少或者根本没有(<1 day 天) |
| 2. Some or a little of the time 不太多(1-2 days 天) |
| 3. Occasionally or a moderate amount of the time 有时或者说有一半的时间(3-4 days天) |
| 4. Most or all of the time 大多数的时间(5-7 days 天) |
| 8. DK 不知道 |
| 9. RF 拒绝回答 |
| **Item 5. I felt hopeful about the future 我对未来充满希望。** |
| 1. Rarely or none of the time 很少或者根本没有(<1 day 天) |
| 2. Some or a little of the time 不太多(1-2 days 天) |
| 3. Occasionally or a moderate amount of the time 有时或者说有一半的时间(3-4 days天) |
| 4. Most or all of the time 大多数的时间(5-7 days 天) |
| 8. DK 不知道 |
| 9. RF 拒绝回答 |
| **Item 6. I felt fearful 我感到害怕。** |
| 1. Rarely or none of the time 很少或者根本没有(<1 day 天) |
| 2. Some or a little of the time 不太多(1-2 days 天) |
| 3. Occasionally or a moderate amount of the time 有时或者说有一半的时间(3-4 days天) |
| 4. Most or all of the time 大多数的时间(5-7 days 天) |
| 8. DK 不知道 |
| 9. RF 拒绝回答 |
| **Item 7. My sleep was restless 我的睡眠不好。** |
| 1. Rarely or none of the time 很少或者根本没有(<1 day 天) |
| 2. Some or a little of the time 不太多(1-2 days 天) |
| 3. Occasionally or a moderate amount of the time 有时或者说有一半的时间(3-4 days天) |
| 4. Most or all of the time 大多数的时间(5-7 days 天) |
| 8. DK 不知道 |
| 9. RF 拒绝回答 |
| **Item 8. I was happy 我很愉快。** |
| 1. Rarely or none of the time 很少或者根本没有(<1 day 天) |
| 2. Some or a little of the time 不太多(1-2 days 天) |
| 3. Occasionally or a moderate amount of the time 有时或者说有一半的时间(3-4 days天) |
| 4. Most or all of the time 大多数的时间(5-7 days 天) |
| 8. DK 不知道 |
| 9. RF 拒绝回答 |
| **Item 9. I felt lonely 我感到孤独。** |
| 1. Rarely or none of the time 很少或者根本没有(<1 day 天) |
| 2. Some or a little of the time 不太多(1-2 days 天) |
| 3. Occasionally or a moderate amount of the time 有时或者说有一半的时间(3-4 days天) |
| 4. Most or all of the time 大多数的时间(5-7 days 天) |
| 8. DK 不知道 |
| 9. RF 拒绝回答 |
| **Item 10. I could not get ”going” 我觉得我无法继续我的生活。** |
| 1. Rarely or none of the time 很少或者根本没有(<1 day 天) |
| 2. Some or a little of the time 不太多(1-2 days 天) |
| 3. Occasionally or a moderate amount of the time 有时或者说有一半的时间(3-4 days天) |
| 4. Most or all of the time 大多数的时间(5-7 days 天) |
| 8. DK 不知道 |
| 9. RF 拒绝回答 |

**Table S2. The 5-item for assessments of life satisfaction, health satisfaction, marital status satisfaction, parent-child relationship satisfaction, and air quality satisfaction.**

| **Life Satisfaction: Please think about your life-as-a-whole. How satisfied are you with it? Are you completely satisfied, very satisfied, somewhat satisfied, not very satisfied, or not at all satisfied?** 总体来看，您对自己的生活是否感到满意？是极其满意，非常满意，比较满意，不太满意，还是一点也不满意？ |
| --- |
| 1. Completely satisfied 极其满意 |
| 2. Very satisfied 非常满意 |
| 3. Somewhat satisfied 比较满意 |
| 4. Not very satisfied 不太满意 |
| 5. Not at all satisfied 一点也不满意 |
| **Health Satisfaction: How satisfied are you with your health?** 您对您的健康满意吗？是极其满意，非常满意，比较满意，不太满意还是一点也不满意？ |
| 1. Completely satisfied 极其满意 |
| 2. Very satisfied 非常满意 |
| 3. Somewhat satisfied 比较满意 |
| 4. Not very satisfied 不太满意 |
| 5. Not at all satisfied 一点也不满意 |
| **Marital satisfaction: How satisfied are you with your marriage (relationship with spouse)?** 您对您的婚姻满意吗？也就是说您对您和您配偶的关系满意吗？是极其满意，非常满意，比较满意，不太满意还是一点也不满意？ |
| 1. Completely satisfied 极其满意 |
| 2. Very satisfied 非常满意 |
| 3. Somewhat satisfied 比较满意 |
| 4. Not very satisfied 不太满意 |
| 5. Not at all satisfied 一点也不满意 |
| 6. No spouse now 现在没有配偶 |
| **Children's relationship satisfaction: How satisfied are you with your relationship with children?** 您对您和您子女的关系满意吗？是极其满意，非常满意，比较满意，不太满意还是一点也不满意？ |
| 1. Completely satisfied 极其满意 |
| 2. Very satisfied 非常满意 |
| 3. Somewhat satisfied 比较满意 |
| 4. Not very satisfied 不太满意 |
| 5. Not at all satisfied 一点也不满意 |
| 6. No child now 现在没有子女 |
| **Air quality satisfaction: How satisfied are you with the air quality this year? Are you completely satisfied, very satisfied, somewhat satisfied, not very satisfied, or not at all satisfied?** 您对今年的空气质量是否感到满意？是极其满意，非常满意，比较满意，不太满意还是一点也不满意？ |
| 1. Completely satisfied 极其满意 |
| 2. Very satisfied 非常满意 |
| 3. Somewhat satisfied 比较满意 |
| 4. Not very satisfied 不太满意 |
| 5. Not at all satisfied 一点也不满意 |

**Table S3.** Pearson’s correlations between different dimensions of satisfaction.

|  | Life satisfaction | Health satisfaction | Marital status satisfaction | Parent-child satisfaction | Air quality satisfaction |
| --- | --- | --- | --- | --- | --- |
| Life satisfaction | 1 |  |  |  |  |
| Health satisfaction | 0.455^**^ | 1 |  |  |  |
| Marital status satisfaction | 0.423^**^ | 0.307^**^ | 1 |  |  |
| Parent-child satisfaction | 0.363^**^ | 0.276^**^ | 0.433^**^ | 1 |  |
| Air quality satisfaction | 0.266^**^ | 0.237^**^ | 0.228^**^ | 0.231^**^ | 1 |

Note: ^*^ *P*<0.05, ^**^ *P*<0.01, ^***^ *P*<0.001

| **Table S4.** Stratified analysis of the association of depressive symptoms with 1-point increase in satisfaction | | | | | | | | | | | | | | | | |
| --- | --- | --- | --- | --- | --- | --- | --- | --- | --- | --- | --- | --- | --- | --- | --- | --- |
| Potential modifiers | Life satisfaction | |  | Health satisfaction | | |  | Marital status satisfaction | |  | Parent-child relationship satisfaction | |  | | Air quality satisfaction | |
|  | OR (95%CI) | *P* |  | OR (95%CI) | *P* |  | | OR (95%CI) | *P* |  | OR (95%CI) | *P* |  | OR (95%CI) | | *P* |
| **Age** |  |  |  |  |  |  | |  |  |  |  |  |  |  | |  |
| <60 | 0.384 (0.357, 0.413) |  |  | 0.433 (0.407, 0.459) |  |  | | 0.546(0.513, 0.581) |  |  | 0.615(0.575, 0.658) | |  | 0.735(0.692, 0.781) | |  |
| ≥60 | 0.403 (0.375, 0.432) | 0.733 |  | 0.435 (0.410, 0.463) | 0.113 |  | | 0.554(0.521, 0.589) | 0.862 |  | 0.620(0.579, 0.664) | 0.390 |  | 0.753(0.709, 0.798) | | 0.422 |
| **Gender** |  |  |  |  |  |  | |  |  |  |  |  |  |  | |  |
| Male | 0.334 (0.308, 0.363) |  |  | 0.377 (0.351, 0.405) |  |  | | 0.495 (0.463, 0.529) |  |  | 0.540 (0.501, 0.581) | |  | 0.637 (0.595, 0.682) | |  |
| Female | 0.405 (0.377, 0.434) | 0.209 |  | 0.446 (0.421, 0.473) | 0.676 |  | | 0.560 (0.526, 0.594) | 0.552 |  | 0.637 (0.596, 0.681) | 0.659 |  | 0.768(0.725, 0.814) | | 0.054 |
| **Residence** |  |  |  |  |  |  | |  |  |  |  |  |  |  | |  |
| Rural | 0.414 (0.385, 0.445) |  |  | 0.459 (0.432, 0.487) |  |  | | 0.579 (0.54, 0.615) |  |  | 0.651 (0.609, 0.696) | |  | 0.786 (0.742, 0.832) | |  |
| Urban | 0.356 (0.331, 0.384) | 0.020 * |  | 0.392 (0.368, 0.418) | 0.227 |  | | 0.505 (0.474, 0.538) | 0.195 |  | 0.569 (0.530, 0.610) | 0.190 |  | 0.677 (0.635, 0.721) | | 0.433 |
| **Educational level** |  |  |  |  |  |  | |  |  |  |  |  |  |  | |  |
| Elementary school or below | 0.408 (0.380, 0.438) |  |  | 0.455 (0.429, 0.482) |  |  | | 0.572 (0.539, 0.607) |  |  | 0.644 (0.602, 0.688) | |  | 0.778 (0.734, 0.824) | |  |
| Middle, high, vocational school and associate degree | 0.349 (0.324, 0.378) | 0.128 |  | 0.382 (0.357, 0.408) | 0.635 |  | | 0.497 (0.465, 0.531) | 0.991 |  | 0.565 (0.526, 0.606) | 0.315 |  | 0.670 (0.628, 0.715) | | 0.104 |
| College and above | 0.253 (0.186, 0.326) | 0.027 * |  | 0.290 (0.212, 0.375) | 0.343 |  | | 0.369 (0.276, 0.468) | 0.682 |  | 0.427 (0.324, 0.535) | 0.192 |  | 0.492 (0.361，0.633) | | 0.311 |
| **Marital status** |  |  |  |  |  |  | |  |  |  |  |  |  |  | |  |
| Married and living with spouse | 0.390 (0.363, 0.419) |  |  | 0.430 (0.405, 0.456) |  |  | | 0.547 (0.514, 0.580) |  |  | 0.613 (0.573, 0.655) | |  | 0.739 (0.697, 0.783) | |  |
| Married but living without spouse | 0.401 (0.366, 0.437) | 0.859 |  | 0.453 (0.418, 0.490) | 0.859 |  | | 0.564 (0.521, 0.609) | 0.167 |  | 0.637 (0.585, 0.690) | 0.641 |  | 0.759 (0.704, 0.819) | | 0.025 ^*^ |
| Single, divorced, and widowed | 0.425 (0.386, 0.467) | 0.359 |  | 0.486 (0.444, 0.531) | 0.629 |  | | 0.583 (0.533, 0.637) | 0.021 * | | 0.661 (0.604, 0.723) | 0.041 |  | 0.804 (0.738, 0.875) | | 0.806 |
| **Smoking Status** |  |  |  |  |  |  | |  |  |  |  |  |  |  | |  |
| Non-smoker | 0.391 (0.364, 0.419) |  |  | 0.432 (0.407, 0.458) |  |  | | 0.547 (0.515, 0.581) | 0.515 |  | 0.666 (0.574, 0.657) | 0.791 |  | 0.739 (0.697, 0.783) | | 0.719 |
| Smoker | 0.423 (0.384, 0.466) | 0.869 |  | 0.467 (0.425, 0.511) | 0.738 |  | | 0.597 (0.549, 0.649) |  |  | 0.614 (0.610, 0.726) | |  | 0.807 (0.741, 0.878) | |  |
| **Drinking Status** |  |  |  |  |  |  | |  |  |  |  |  |  |  | |  |
| Non-drinker | 0.397 (0.370, 0.426) |  |  | 0.436 (0.410, 0.462) |  |  | | 0.556 (0.523, 0.590) | 0.929 |  | 0.624 (0.584, 0.667) | 0.324 |  | 0.750 (0.708, 0.795) | | 0.102 |
| Drinker | 0.374 (0.346, 0.405) | 0.357 |  | 0.426 (0.398, 0.456) | 0.407 |  | | 0.527 (0.493, 0.564) |  |  | 0.595 (0.553, 0.640) | |  | 0.718 (0.672, 0.767) | |  |
| **Regional categories** |  |  |  |  |  |  | |  |  |  |  |  |  |  | |  |
| East | 0.351 (0.325, 0.378) |  |  | 0.380 (0.356, 0.405) |  |  | | 0.495 (0.464, 0.528) |  |  | 0.560 (0.522, 0.600) | |  | 0.666 (0.626, 0.708) | |  |
| Midland | 0.404 (0.375, 0.434) | 0.424 |  | 0.449 (0.422, 0.477) | 0.026 ^*^ |  | | 0.564 (0.529, 0.600) | 0.526 |  | 0.636 (0.594, 0.680) | 0.177 |  | 0.764 (0.719, 0.811) | | 0.827 |
| West | 0.436 (0.405, 0.470) | 0.008** |  | 0.487 (0.457, 0.519) | 0.001 ^**^ |  | | 0.606 (0.568, 0.646) | 0.293 |  | 0.671 (0.625, 0.720) | 0.946 |  | 0.824 (0.775, 0.876) | | 0.692 |

Note: ^*^ *P*<0.05, ^**^ *P*<0.01, ^***^ *P*<0.001
